# Immuno-oncological interactions between meningeal lymphatics and glioblastoma: from mechanisms to therapies

**DOI:** 10.7150/thno.111972

**Published:** 2025-06-09

**Authors:** Nan Wen, Xiao Xiao, Huangjie Lu, Qingyuan Chen, Genghong He, Zhiyuan Qian, Jianfeng Zeng, Li Xiao

**Affiliations:** 1 The Second Affiliated Hospital of Soochow University, Suzhou 215004, China.; 2 Center for Molecular Imaging and Nuclear Medicine, State Key Laboratory of Radiation Medicine and Protection, School for Radiological and Interdisciplinary Sciences (RAD-X), Collaborative Innovation Center of Radiological Medicine of Jiangsu Higher Education Institutions, Soochow University, Suzhou 215123, China.; 3 Department of Radiology, The First Affiliated Hospital of Soochow University, Suzhou 215006, China.

**Keywords:** central nervous system, meningeal lymphatic vessels, glymphatic system, immune modulation, glioblastoma, tumor therapy

## Abstract

The recent discovery of meningeal lymphatic vessels (MLVs) has revolutionized our understanding of immune regulation within the central nervous system (CNS), overturning the long-standing view of the brain as an immune-privileged organ. Glioblastoma (GBM), the most aggressive primary brain tumor, remains therapeutically intractable due to its highly immunosuppressive microenvironment and poor response to conventional and immune-based therapies. Emerging evidence suggests that MLVs play a crucial role in CNS immune surveillance, cerebrospinal fluid drainage, and solute clearance, all of which are directly linked to GBM pathophysiology. This review is motivated by the urgent need to explore novel therapeutic strategies that address GBM's immune escape and therapeutic resistance. We comprehensively analyze the bidirectional interactions between MLVs and GBM, including their role in antigen transport, T cell activation, and tumor dissemination. Furthermore, we evaluate the therapeutic potential of targeting MLVs through lymphangiogenic stimulation or as alternative routes for immune modulation and drug delivery. These approaches offer promising avenues to enhance anti-tumor immunity and may pave the way for next-generation treatment paradigms in GBM.

## 1. Introduction

Glioblastoma (GBM) is the most aggressive primary brain tumor, classified as a World Health Organization (WHO) grade 4 infiltrative glioma 1. Despite advances in neurosurgical techniques and adjuvant therapies, the prognosis for GBM remains dismal, with a median overall survival of only 14.6 months and a five-year survival rate of just 5.4% 2-5. The challenges in GBM management stem from its cranial hypertension, extensive heterogeneity, and profound immune escape mechanisms, all of which contribute to therapeutic resistance and high recurrence rates 6-10.

The central nervous system (CNS) has long been considered “immunologically privileged”, with limited immune surveillance due to the blood-brain barrier (BBB) and the absence of a well-defined lymphatic system 11-14. However, the discovery of meningeal lymphatic vessels (MLVs) has reshaped this perspective. MLVs, located in the dura mater near the dural sinuses, serve as critical pathways for cerebrospinal fluid (CSF) drainage and immune cell trafficking, connecting the CNS to peripheral lymphatic networks 15, 16. These vessels play essential roles in maintaining CNS homeostasis, facilitating waste clearance, and regulating immune responses.

In the context of GBM, MLVs offer a promising yet underexplored avenue for modulating the tumor's immunosuppressive microenvironment. Emerging evidence suggests that MLVs are involved in transporting tumor antigens to the deep cervical lymph nodes, thereby contributing to peripheral immune activation and potential anti-tumor responses 17. At the same time, lymphangiogenesis has been implicated in tumor progression in several extracranial cancers. Although direct evidence for MLV-mediated tumor cell dissemination in GBM is lacking, the remodeling of MLVs in glioma models raises important questions about their role in shaping tumor-immune dynamics 18, 19.

Given the limited success of current immunotherapies, such as immune checkpoint inhibitors, targeting MLVs represents an innovative therapeutic strategy in treating GBM 20, 21. By enhancing lymphatic drainage and modulating immune responses, MLVs could overcome some of the key barriers in GBM treatment. This review delves into the emerging field of MLV research, focusing on their immuno-oncological interactions with GBM. We aim to provide a comprehensive overview of the mechanisms linking MLVs to GBM pathophysiology and evaluate the potential of MLV-targeted therapies in advancing GBM treatment.

## 2. Pathophysiology and key clinical challenges of GBM

**Figure 1:**
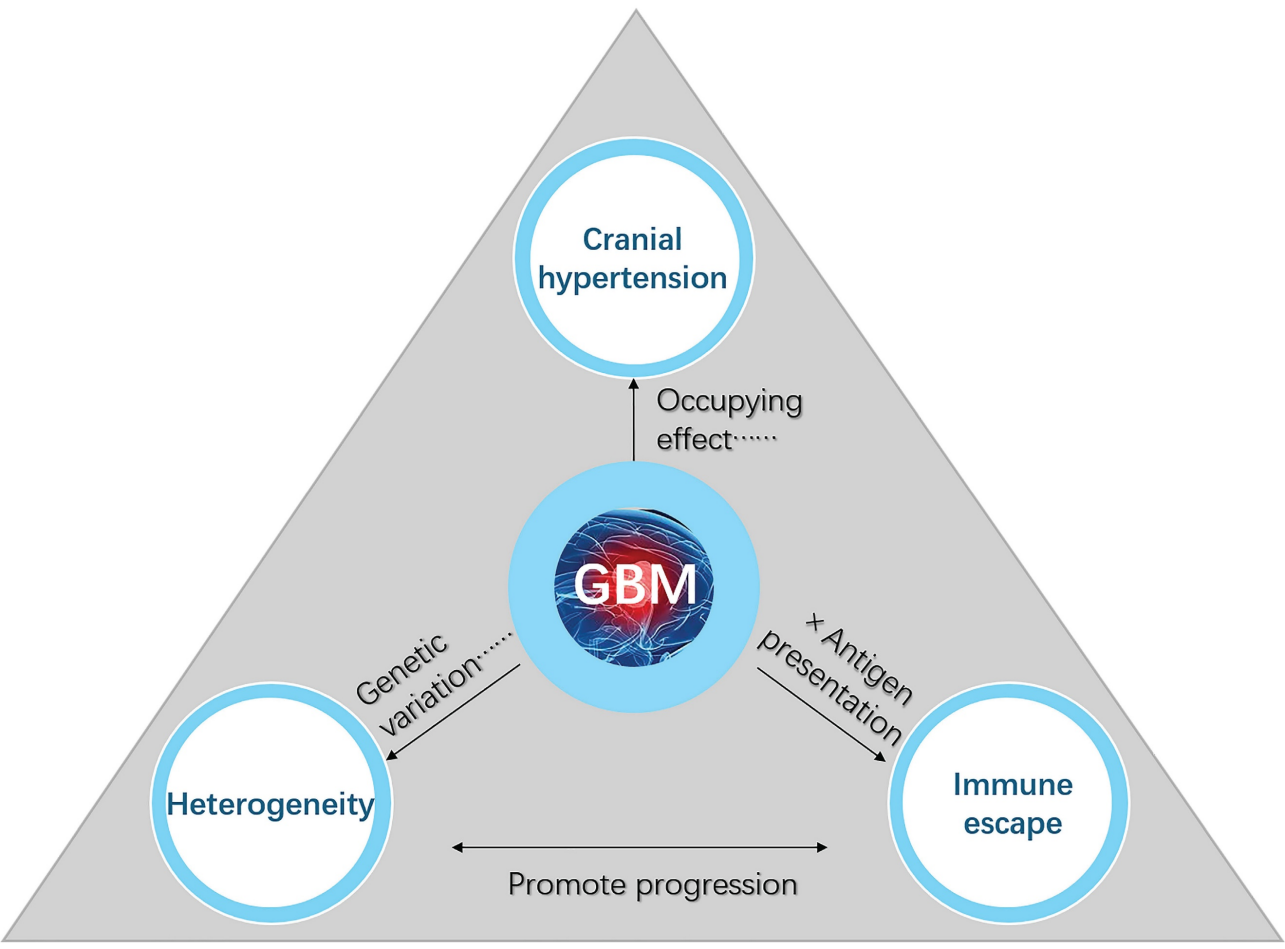
** Key factors contributing to the poor prognosis of GBM.** Cranial hypertension, tumor heterogeneity, and immune escape are three interconnected factors that significantly contribute to the poor prognosis and therapeutic resistance of GBM. Cranial hypertension arises from tumor-induced space occupation and peritumoral edema, compounded by impaired cerebrospinal fluid outflow, which exacerbates intracranial pressure and disrupts normal brain function. Tumor heterogeneity, driven by glioblastoma stem cells and the tumor microenvironment, promotes invasive growth, metastasis, and treatment resistance through diverse cellular phenotypes and genetic variations. Immune escape mechanisms, including limited antigen presentation, T-cell infiltration barriers, and tumor-associated macrophage polarization toward the immunosuppressive M2 phenotype, further reduce the efficacy of immune-based therapies. Together, these interlinked factors disrupt immune regulation, impair waste clearance, and synergistically drive GBM progression and hinder therapeutic success.

GBM is a highly aggressive brain tumor characterized by its rapid growth, extensive molecular and cellular heterogeneity, and profound disruptions to intracranial and systemic physiological processes 22, 23. Despite advancements in surgical techniques, radiation therapy, and pharmacological interventions, the prognosis for GBM remains poor, largely due to its unique and multifaceted challenges 3, 24, 25. As GBM progresses within the confined cranial cavity, it exerts significant disruptions to structural and functional homeostasis, including increased intracranial pressure, tumor-induced heterogeneity, and immune escape mechanisms. These interrelated factors not only contribute to the tumor's aggressive nature but also limit the efficacy of existing treatments (Figure 1).

### 2.1 Cranial hypertension

Clinically, the majority of GBM patients experience varying degrees of cranial hypertension as the disease progresses, significantly impairing their quality of life. The mechanism underlying glioma-induced intracranial hypertension is intricate, primarily attributed to the interplay of multiple factors, including the tumor's space-occupying effect, cerebrospinal fluid circulation disturbances, peritumoral edema, and secondary pathological alterations.

Unlike many other malignancies that grow in compliant tissues, GBM develops within the rigid confines of the skull. As the tumor expands, it compresses surrounding brain parenchyma and consumes limited intracranial space, leading to direct increases in intracranial hypertension. Moreover, GBM interferes with CSF dynamics. CSF is primarily produced by the choroid plexus located in the lateral ventricles and flows sequentially through the third and fourth ventricles before being absorbed by the arachnoid granulations into the cerebral venous sinuses to maintain circulation within the brain 26, 27. However, during the development process of glioma, tumor growth may block these CSF flow pathways, such as the cerebral aqueduct or the fourth ventricle, leading to obstructive hydrocephalus 28, 29. Alternatively, the tumor may impair CSF absorption by damaging arachnoid granulations, resulting in communicating hydrocephalus. Additionally, compression of cerebral veins or dural venous sinuses can hinder venous outflow, causing venous congestion, increased intracranial blood volume, and even thrombosis 30. Some gliomas also exhibit a tendency toward intratumoral hemorrhage or cyst formation due to vascular fragility, which can cause acute increases in tumor volume and intracranial hypertension 31, 32.

The hypervascular nature of GBM introduces further complications. Neovessels formed within the tumor are often structurally abnormal and excessively permeable due to BBB disruption, leading to peritumoral vasogenic edema that exacerbates intracranial pressure. Compounding this, the typically low expression of lymphangiogenic factors such as vascular endothelial growth factor-C (VEGF-C) in GBM restricts lymphatic drainage of CSF and metabolic waste, contributing further to pressure elevation and fluid retention 33-35. Collectively, these factors establish a vicious cycle in which elevated intracranial pressure impairs neurological function and further worsens the tumor microenvironment, presenting significant barriers to effective treatment. While temporary relief can be achieved through measures such as CSF diversion or osmotic diuretics 10, 36, intracranial hypertension remains a persistent and complex clinical challenge.

### 2.2 Heterogeneity of GBM

GBM is characterized by significant inter- and intra-tumor heterogeneity, which arises from intricate interactions across multiple levels, including genetic variations, epigenetic modifications, cellular origins, and microenvironmental regulation. This heterogeneity underpins the aggressive behavior, treatment resistance, and recurrence of GBM 37, 38. At the molecular level, genetic heterogeneity plays a central role. Distinct tumor subclones may harbor unique driver mutations, such as IDH1/2 mutations, EGFR amplification, or TP53 deletion, which influence proliferation, metabolism, and differentiation 39-41. Epigenetic mechanisms, such as DNA methylation and histone modification, further contribute to phenotypic diversity. For instance, MGMT promoter methylation is associated with reduced responsiveness to temozolomide (TMZ) 42, while promoter methylation of lncRNAs (e.g., CD109-AS1, LINC02447) has been linked to immune escape and tumor progression 43. In addition, global DNA methylation abnormalities or histone modification differences (e.g., H3K27me3) can cause cells with identical genotypes to exhibit diverse phenotypes, such as mesenchymal transformation, thereby enhancing tumor adaptability and complexity 44, 45.

Beyond inherent molecular and genetic alterations, GBM heterogeneity is also shaped by its tumor microenvironment (TME). In hypoxic regions, the upregulation of programmed death ligand-1 (PD-L1) via HIF-1α activation leads to metabolic reprogramming, such as angiogenesis induction (e.g., high VEGF expression) and enhanced glycolysis 46, 47. Tumor cells interact with surrounding stromal cells by transferring factors like TGF-β through exosomes, promoting invasiveness and treatment resistance 48, 49. Furthermore, the metabolic diversity between tumor core and periphery, including shifts between glycolysis, oxidative phosphorylation, and lipid metabolism, adds further complexity to therapeutic targeting 50.

**Figure 2:**
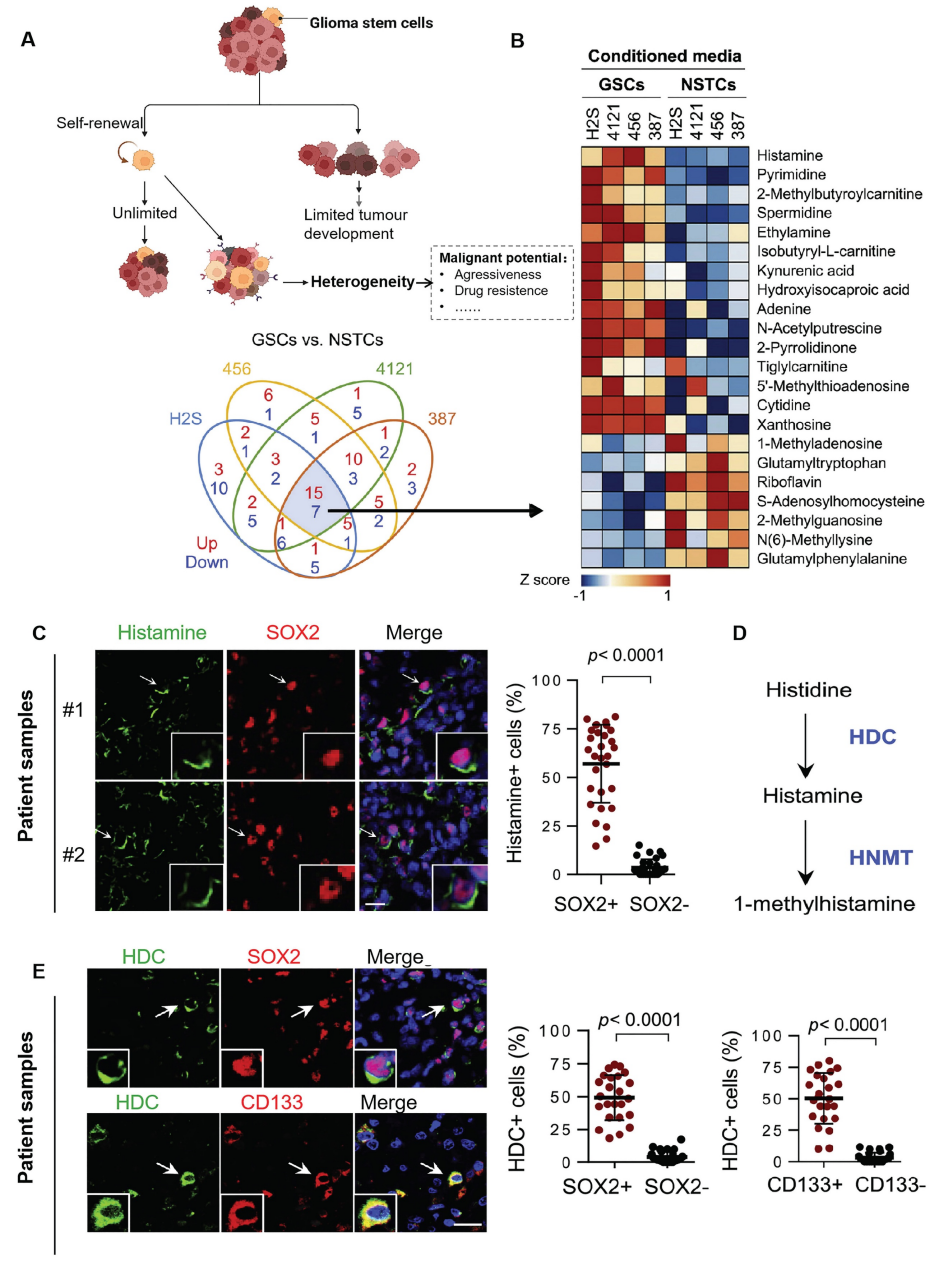
** Tumor stem cells can confer multiple heterogeneities to tumor cells. (A)** As stem cells, GSCs act with the ability to self-renew and differentiate. It can also give other heterogeneous characteristics to other tumor cells, thus increasing the invasiveness of GBM, promoting proliferation and metastasis, and even developing immunosuppressive properties. **(B)** Heatmap of mass spectrometric analysis of secreted metabolites of 4 GSC cells versus paired non-stem tumor cells (NSTCs). Red indicates upregulated metabolites and blue indicates downregulated ones. **(C)** Immunofluorescence analysis of histamine and SOX2 in 6 GBM samples. Left: representative image; Scale bar, 20 μm. Right: percentage of histamine-positive cells in SOX2-positive cells compared to SOX2-negative cells by t-test in five randomly selected microscopic fields of view for each tumor. **(D)** Metabolic pathway of histamine. Metabolic enzymes are blue. HDC: histidine decarboxylase; HNMT: histamine N-methyltransferase. **(E)** Immunofluorescence analysis of HDC, SOX2, and CD133 in 6 GBM samples; Left: representative images; Scale bars, 20 μm. Right: comparison of the percentage of HDC-positive cells in SOX2- or CD133-positive versus SOX2- or CD133-negative cells by t-test in five randomly selected microscopic fields of view for each tumor. Reproduced with permission from Ref. 56. Copyright 2022, Elsevier Cell Stem Cell.

Furthermore, GBM arises from diverse cellular lineages, including neural stem cells and oligodendrocyte precursor cells 51-53. Among these, glioma stem cells (GSCs) are pivotal in generating heterogeneous cell populations through environment-dependent differentiation 53-55. GSCs are highly resistant to therapy and a key source of recurrence. They actively shape the TME by secreting metabolites such as histamine and nucleotides that promote tumor progression (Figure 2) 56. Tumor-associated macrophages (TAMs), which comprise up to 40% of the tumor mass, also influence heterogeneity. Derived from both microglia and bone marrow myeloid cells, TAMs interact closely with GSCs, with their density and phenotype tightly linked to GSC activity 57.

### 2.3 Immune escape of the CNS

Under normal physiological conditions, immune activity in the healthy brain remains minimal or in a quiescent state, with microglia expressing low levels of major histocompatibility complex (MHC) molecules to maintain immune privilege 58. However, in GBM, this balance is disrupted, giving rise to an immunosuppressive and heterogeneous TME. This state is driven by complex interactions among tumor cells, immune cells, stromal elements, and secreted factors.

**Figure 3:**
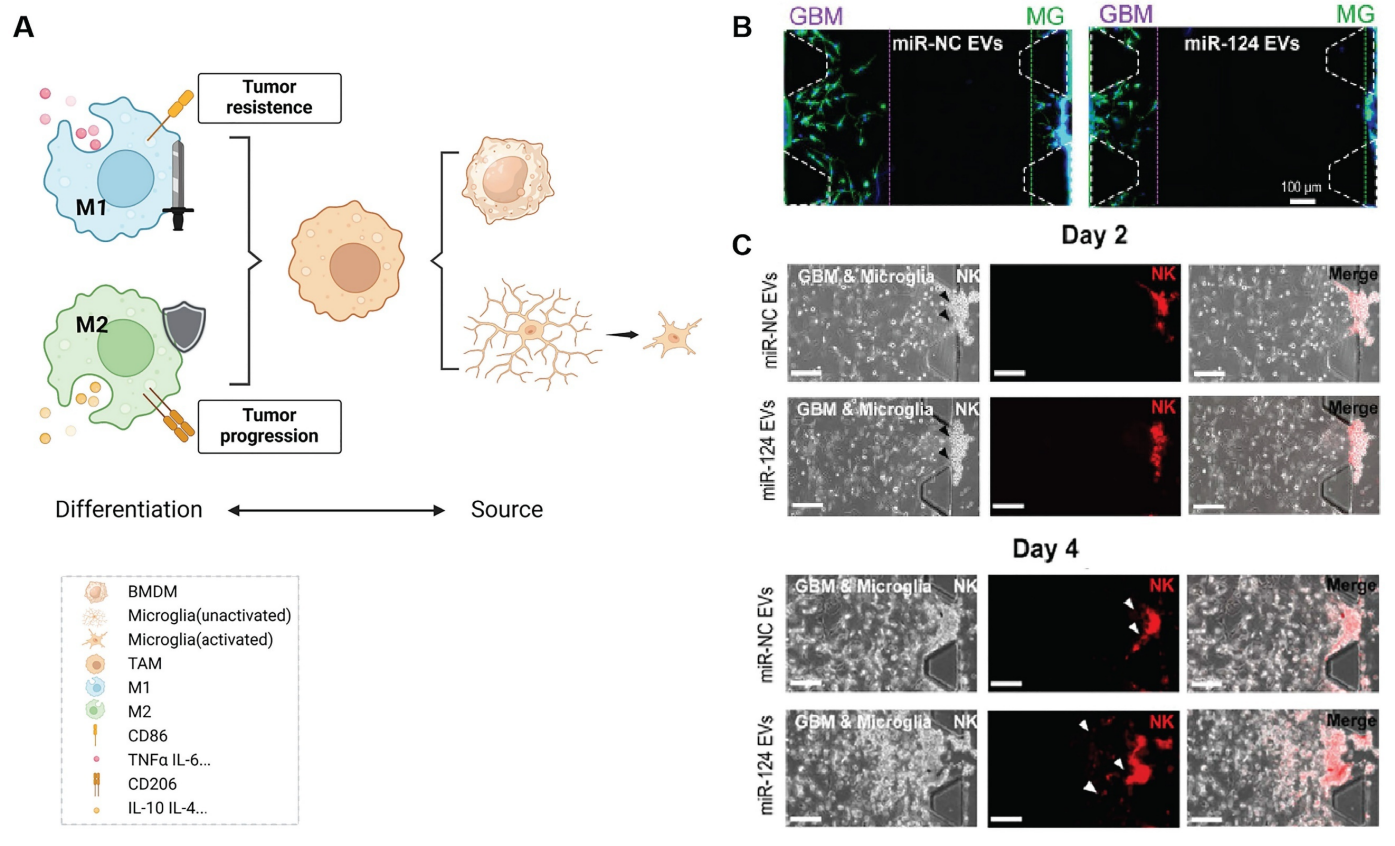
** Inhibition of M2-type TAM activation suppresses GBM progression. (A)** TAMs consist of two main sources: microglia and bone marrow-derived macrophages (BMDMs), the latter constituting over 90% of TAMs in GBM. TAMs can differentiate into either M1 or M2 phenotypes upon activation, with M1 TAMs exhibiting anti-tumor properties and M2 TAMs promoting immunosuppression and tumor progression. **(B)** Co-culture of U373MG GBM cells and microglia with miRNA EVs showed that miR-124 EV treatment significantly inhibited cell migration compared to miR-NC EV treatment. Immunostaining for F-actin (green) and nuclei (blue) revealed shorter maximum migration distances of both GBM and microglial cells toward the gel in the miR-124-treated group, indicating reduced migratory capacity. **(C)** In a microfluidic device, U373MG and microglia (embedded in collagen gels at a 2:1 ratio) were co-cultured for 2 days prior to the introduction of NK cells. Representative images of NK cells immunostained with PE-coupled CD45 (red) on day 2 and day 4 showed increased NK cell infiltration in the miR-124 EV-treated system compared to the miR-NC control. Reproduced with permission from Ref. 62. Available under a CC-BY 4.0 license. Copyright 2021, The author(s).

GBM promotes immune escape by recruiting immunosuppressive cell types such as regulatory T cells (Tregs), myeloid-derived suppressor cells (MDSCs), and M2-polarized tumor-associated macrophages 59. Among these, microglia and bone marrow-derived macrophages play central roles in modulating immune responses. As the tumor progresses, macrophages acquire immunosuppressive phenotypes that support tumor growth and angiogenesis 60. In particular, hypoxic and necrotic regions of GBM secrete chemokines such as interleukin-4 (IL-4) and interleukin-10 (IL-10), driving a shift from M1 (anti-tumor) to M2 (pro-tumor) TAMs. This shift facilitates immune tolerance, promotes tumor invasion, and enhances angiogenesis (Figure 3A). M2 TAMs directly inhibit the proliferation and cytotoxic function of effector T cells through secretion of IL-10, TGF-β1, and other cytokines. Simultaneously, they suppress the secretion of pro-inflammatory cytokines such as interferon-gamma (IFN-γ), weakening the immune response and further promoting tumor progression 61.

Given the pivotal role of TAM polarization in establishing an immunosuppressive microenvironment, reversing M2 dominance has emerged as a therapeutic strategy. The Hong *et al.* 62 employed HEK293T cell-derived exosomes to deliver miR-124, an inhibitor of M2 polarization, into glioma cells (U373MG). This intervention reduced tumor cell migration and invasiveness (Figure 3B) and promoted natural killer (NK) cell infiltration (Figure 3C). In addition, the exosome LINC01232 derived from M2 can induce immune escape in glioma. LINC01232 binds to E2F2 to enter the nucleus and collaboratively upregulates NBR1, mediating the degradation of histocompatibility complex Class I molecules (MHC-I) by autophagy lysosomes, enabling CD8^+^ T cells to effectively recognize tumor-specific antigens and thereby evade immune surveillance. When E2F2/NBR1 is inhibited or LINC01232 is knocked out, the expression of MHC-I on the surface of tumor cells can be restored and the therapeutic effect of T cells can be enhanced 7.

Abnormal activation of immune checkpoint molecules exacerbates immunosuppression. For example, glioma cells overexpress PD-L1, which binds to PD-1 on T cells, inducing T cell exhaustion or apoptosis. Other checkpoint molecules, including CTLA-4 and TIM-3, may also cooperatively inhibit T cell function, forming multiple pathways for immune escape 63-68. The presence of GSCs further enhances immune escape by secreting ZNF16 via exosomes, which binds to the TGF-β promoter in normal human astrocytes (NHAs), activating the TGF-β pathway. This reprograms NHAs into tumor-associated astrocytes (TAAs), thereby enhancing proliferation and migration capabilities and contributing to the invasiveness and chemoresistance of glioblastoma 69. These mechanisms collectively form a dynamic immunosuppressive network, allowing glioma to continuously evade immune attack and contributing to its malignant progression and treatment resistance.

In addition to cellular-level immune regulation, the immune microenvironment of GBM is also regulated by the systemic immune system. The lymphatic system, which plays a crucial role in maintaining osmotic balance and enabling immune surveillance in normal tissues, serves as a key channel for the circulation of immune cells and factors. Under physiological conditions, immune cells such as T cells, macrophages, neutrophils, dendritic cells (DCs), and B cells accumulate in the meningeal interstitium, where they perform immune surveillance. The meninges also regulate T cell infiltration into the CNS, contributing to the brain's unique immune privilege 70, 71. However, the recent discovery of meningeal lymphatics has challenged traditional views of CNS immunity. These lymphatic vessels connect CSF circulation with peripheral immune pathways, creating a link between the brain and the systemic immune system. In the context of GBM, meningeal lymphatics may play a more complex role by influencing immune cell trafficking, modulating cranial hypertension, and contributing to the heterogeneity of the tumor microenvironment 20. Understanding these connections could reveal new immuno-oncological mechanisms underlying GBM progression and therapeutic resistance.

## 3. Meningeal lymphatic vessels

### 3.1 Historical background and discovery of meningeal lymphatic vessels

**Figure 4:**
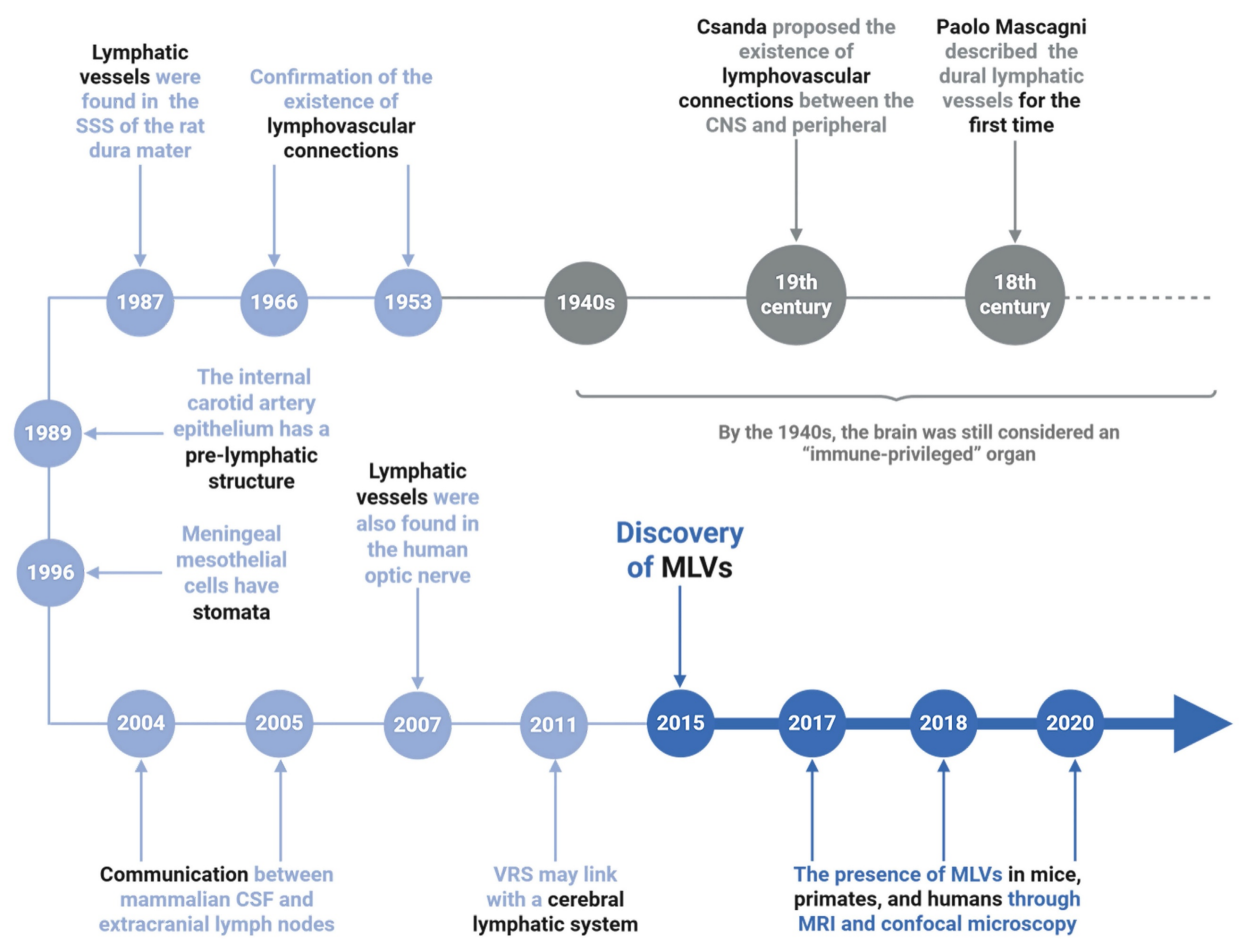
** The discovery process of meningeal lymphatic vessels.** The timeline traces key discoveries in MLVs research. Early hints emerged in the 1940s-1980s, including lymphatic-like structures in the rat dura mater (1966) and unique features of carotid artery epithelium (1987). The 1990s-2000s revealed meningeal mesothelial cell properties. Breakthroughs from 2004-2020 confirmed functional MLVs in mice and humans via MRI/confocal microscopy, with findings extending to the human optic nerve and potential glymphatic system connections. These milestones established MLVs as critical players in brain waste clearance and neuroimmunology, reshaping understanding of neurological diseases.

The existence of lymphatic structures within the cranial compartment has long been debated. As early as the late 18th century, the Italian anatomist Paolo Mascagni provided anatomical illustrations suggesting lymphatic-like vessels in the dura mater 72, 73. In the 19th century, Csanda proposed possible lymphatic connections between the CNS and peripheral circulation 74, 75. However, the dominant paradigm for much of the 20th century, largely shaped by Peter Medawar's concept of the brain as an “immune-privileged organ” posited that the CNS lacked functional lymphatic drainage 74-78. Despite early speculations, definitive structural evidence for intracranial lymphatics remained elusive until the latter half of the 20th century 79, 80. In 1987, Andres *et al.* 81 identified the presence of lymphatic vessels in the wall of the superior sagittal sinus (SSS) of the rat dura mater by electron microscopy. Wang *et al.* 82 subsequently described pre-lymphatic structures along the internal carotid and vertebrobasilar arteries that appeared to facilitate fluid drainage to extracranial deep cervical lymph nodes (dCLNs). Additional ultrastructural studies by Li *et al.* 83 and immunostaining of human optic nerves using the lymphatic marker D2-40 84 provided further circumstantial evidence for CNS-associated lymphatic networks. Pioneering work by Johnston *et al.* 85 and Gao *et al.* 86 demonstrated CSF outflow pathways connecting to extracranial lymphatic systems, challenging prior notions of CNS isolation. Marín-Padilla *et al.* 87 proposed that the perivascular (Virchow-Robin) spaces might function as components of an intracerebral pre-lymphatic network, further supporting the idea of fluid clearance beyond the CNS parenchyma. A major breakthrough occurred in 2015, when definitive evidence of functional lymphatic vessels in the dura mater was provided independent by Louveau *et al.* and Aspelund *et al.* 16. Tracers injected into the brain parenchyma were detected in the ipsilateral dCLNs, and downstream occlusion of lymphatic flow led to upstream dilation of dural vessels. These findings provided the first direct demonstration of a functional meningeal lymphatic system. Subsequent anatomical and imaging studies have mapped the distribution of MLVs along the transverse sinus, anterior and middle meningeal arteries in mice, and similar structures have since been confirmed in primates and humans using confocal microscopy and high-resolution magnetic resonance imaging (MRI) 88-91. In humans, MLVs show strong anatomical and functional connectivity with dCLNs, suggesting active participation in CSF drainage and immune surveillance (Figure 4) 92.

### 3.2 Structure and function characteristics of meningeal lymphatic vessels

Lymphatic vessels are broadly categorized into initial lymphatic vessels and collecting lymphatic vessels, each serving distinct roles in fluid drainage and immune regulation. Initial lymphatics are thin-walled structures formed by a single layer of lymphatic endothelial cells, which are highly permeable and essential for lymphangiogenesis 93, 94. These vessels lack smooth muscle cells (SMCs) and have discontinuous basement membranes connected by button-like junctions, allowing the entry of interstitial fluid (ISF), macromolecules, and immune cells through primary lymphatic valves 78, 95. In contrast, collecting lymphatics possess smooth muscle layers, secondary valves, and continuous “zipper-like” junctions, facilitating unidirectional lymph flow and preventing reflux 96.

Building on this general framework, MLVs are anatomically divided into basal MLVs and dorsal subsets, each exhibiting distinct anatomical and functional characteristics. Dorsal MLVs travel along the SSS and transverse sinus (TS), are smaller in diameter, lack lymphatic valves, and exhibit discontinuous vascular structures. These vessels are morphologically underdeveloped and primarily follow the dural folds and venous sinuses. While initially hypothesized to facilitate macromolecule clearance, recent studies indicate that dorsal MLVs are structurally unsuited for bulk fluid or large-molecule drainage, limiting their contribution under physiological conditions 15, 97-99. In contrast, basal MLVs, positioned near the skull base, exhibit larger lumens, extensive branching, and functional valves. Their lymphatic endothelial cells (LECs) display oak-leaf-shaped nuclei and button-like junctions, enabling efficient uptake of CSF and large solutes. These vessels form cistern-like expansions that promote fluid pooling and clearance toward the dCLNs, closely resembling peripheral collecting lymphatics 16.

**Figure 5:**
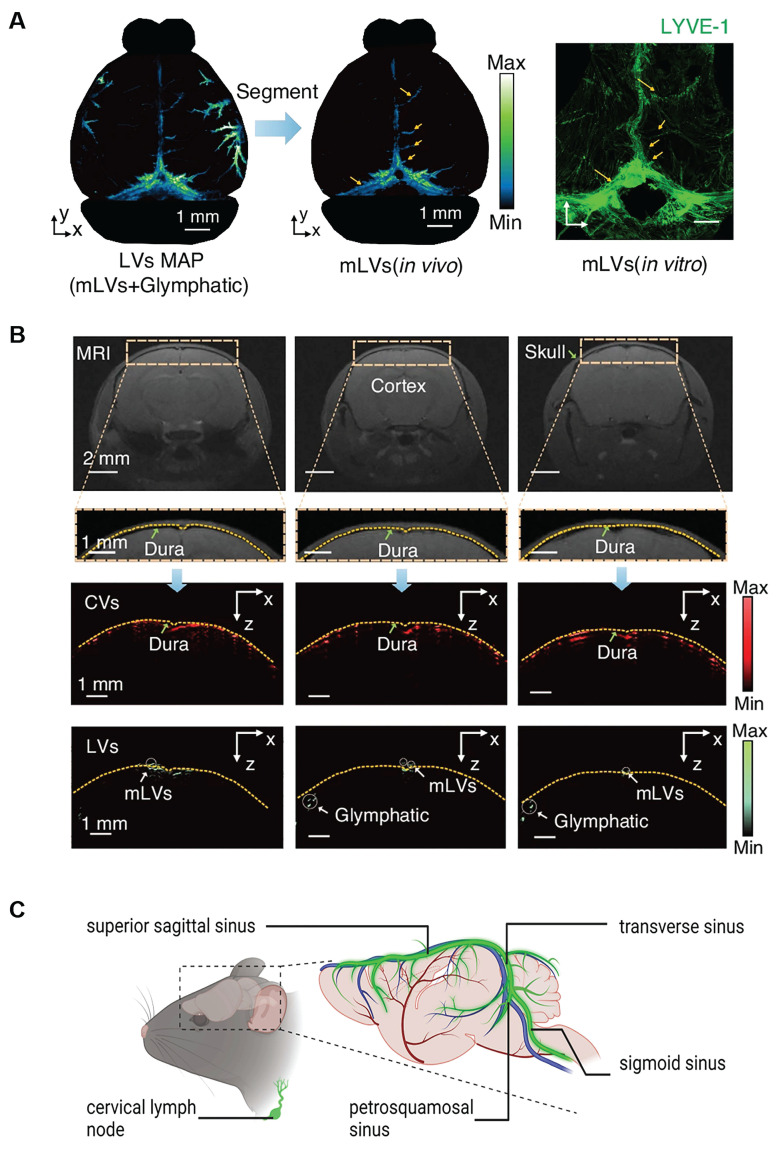
** Anatomy and location of basal and dorsal MLVs. (A)** Photoacoustic imaging reveals the stereoscopic morphology of mouse MLVs, demonstrating depth layering within a range of approximately 3.75 mm (scale bar: 1 mm). LYVE-1 staining further confirms the structural characteristics of MLVs *in vitro*. **(B)** Magnetic resonance imaging of coronal views of cerebral vessels and lymphatic vessels supports the presence of MLVs surrounding the transverse sinus and superior sagittal sinus. Fluorescence imaging further confirms their anatomical localization in the dura mater. Reproduced with permission from Ref. 100. Available under a CC-BY 4.0 license. Copyright 2024, Nature light: science & applications. **(C)** Schematic illustration of mouse meningeal lymphatic vessels and their anatomical course. These vessels accompany major veins, including those along the sigmoid sinus and transverse sinus, and extend toward the cervical lymph nodes.

Imaging studies have further clarified the anatomical and functional distinctions between basal and dorsal MLVs. Photoacoustic imaging (Figure 5A) and fluorescence imaging reveals the localization of MLVs within the dura mater, with dorsal MLVs aligning along the SSS TS, and basal MLVs positioned at the skull base. MRI scans (Figure 5B) demonstrate the route of CSF outflow, tracing its movement from the cisterna magna through basal MLVs toward cervical lymphatic structures 15,100. Based on these data, a schematic anatomical map of the mouse meningeal lymphatic system has been reconstructed (Figure 5C). Further evidence comes from fluorescent stereomicroscopy performed in Prox1-GFP transgenic mice following the injection of PEG-IRDye into the brain parenchyma. In control animals, lymphatic tracer was observed migrating toward the dCLNs. However, in mice where the efferent lymphatic vessels of the dCLNs were surgically ligated, upstream MLVs became markedly dilated, particularly on the ipsilateral side, indicating obstructed drainage. In contrast, superficial cervical lymph nodes (sCLNs) did not display significant changes following ligation, suggesting that dCLNs serve as the primary outflow route for brain-derived lymphatic fluid 99. These findings reinforce the notion that basal MLVs are the primary conduits for CSF outflow and macromolecular clearance, while dorsal MLVs may contribute only minimally to active drainage under normal physiological conditions 15.

## 4. Dual roles of MLVs in glioblastoma immuno-oncology

### 4.1 Development and formation of MLVs

MLVs Lymphatic vessels are composed of LECs, which are differentiated from venous endothelial cells and begin to develop at 6 to 7 weeks in the human embryo and approximately 9.5 to 10.5 days in the mouse embryo 101. During maturation, LECs express a suite of canonical lymphatic markers, including Lyve-1, Prox1, PDPN, and VEGFR3 16, 102. MLVs first emerge near the skull base and progressively expand postnatally to cover the entire meningeal compartment. In mice, these vessels extend from the cribriform plate adjacent to the olfactory bulbs to the caudal spinal meninges, reaching the lumbar region 98, 99, 103-106. Connections of LECs require anchoring filaments (composed of fibulin-1, emilin-1, and integrin α9β1) that link the extracellular matrix to the cytoskeleton, along with tight junction proteins (Occludin, Claudin-5, ZO-1) and adhesion molecules (ESAM, JAM-A). These, together with lymphoid markers like PECAM-1 (CD31) and Lyve1, enable dynamic regulation of lymphatic drainage and immune cell trafficking 96, 107-110.

Lymphangiogenesis, the process of lymphatic vessel formation, involves LEC proliferation, migration, and tube morphogenesis. This is primarily driven by VEGF-C binding to its receptor VEGFR3. In pathological contexts (e.g., inflammation and tumors), VEGF-C expression is markedly upregulated to promote neolymphangiogenesis, although physiological triggers such as physical activity and adipose remodeling can also stimulate this pathway 96, 111-113. In murine models, inhibition of VEGFR3 during development results in significant MLV regression, indicating its critical role in embryonic lymphangiogenesis. In adult tissues, certain lymphatic beds retain VEGFR3 dependency for maintenance and remodeling 114, 115.

### 4.2 The role of MLVs in glioblastoma immunity

MLVs are critical players in glioblastoma immunity, facilitating tumor drainage and modulating immune responses. By transporting tumor cells and antigens to deep cervical lymph nodes, MLVs enable dendritic cells to present tumor antigens and activate T cells 16, 20. Preclinical studies 116 in GBM mouse models have demonstrated that exogenous VEGF-C promotes meningeal lymphangiogenesis, enhancing CD8^+^ T cell activation and migration to tumor sites. This leads to persistent anti-tumor immune responses and significantly improves survival rates in treated mice. Furthermore, VEGF-C, when combined with immune checkpoint inhibitors, produces synergistic anti-tumor effects, underscoring the therapeutic potential of targeting MLVs.

The functional integrity of MLVs has been shown to correlate with the efficacy of GBM therapies. MLVs enhance tumor immune surveillance by promoting lymphocyte infiltration and activating specific T cell responses, ultimately slowing tumor growth 104. Conversely, disruption of MLVs or removal of dCLNs reduces DC-mediated drainage and CD8^+^ T cell activation, leading to diminished therapeutic efficacy and decreased survival rates in GBM models 17. These findings underscore the importance of MLVs in orchestrating anti-tumor immunity in GBM. Further exploration of their role in immune activation may help refine strategies to improve the efficacy of GBM immunotherapies.

### 4.3 Tumor-induced remodeling and pro-tumor effects of MLVs

Tumor-associated lymphangiogenesis is a well-established feature in several extracranial cancers, where it often correlates with increased vessel permeability and metastatic potential 111, 117, 118. In the CNS, the expression of lymphangiogenic factors such as VEGF-C/D, PDPN, and VEGFR3 is also upregulated in malignant gliomas, particularly in recurrent tumors, raising the possibility that MLV remodeling may occur in response to tumor-driven stimuli 119-121.

Although most clinical evidence in neuroblastoma and melanoma suggests a link between VEGF-C signaling and lymphatic dissemination, the relevance of these findings to GBM remains to be definitively established. Preclinical studies have shown that VEGF-C expression in glioma models can stimulate meningeal lymphangiogenesis and potentially enhance immune response to therapies such as anti-PD-1/CTLA-4 checkpoint inhibitors 104. Conversely, blockade of the CCL21-CCR7 axis abrogated this therapeutic benefit, implying a role for MLV remodeling in facilitating immune cell trafficking.

Interestingly, dorsal MLVs appear more susceptible to tumor-induced structural remodeling than basal MLVs 104, 116. This remodeling is characterized by altered vessel diameter, branching patterns, and transcriptional upregulation of lymphangiogenesis-related genes. However, there is currently no direct evidence that MLV remodeling in glioma facilitates true lymphatic metastasis. The potential for tumor cells to migrate via MLVs may involve adhesion molecules, integrin signaling, or ECM degradation by matrix metalloproteinases (MMPs), but such mechanisms remain speculative in the context of GBM 122, 123. Therefore, while tumor-induced lymphangiogenesis and MLV remodeling in GBM may enhance immunotherapy efficacy, their pro-metastatic potential remains unproven. Further studies are needed to determine whether MLVs can serve as conduits for tumor dissemination or merely act as immunological modulators.

**Figure 6:**
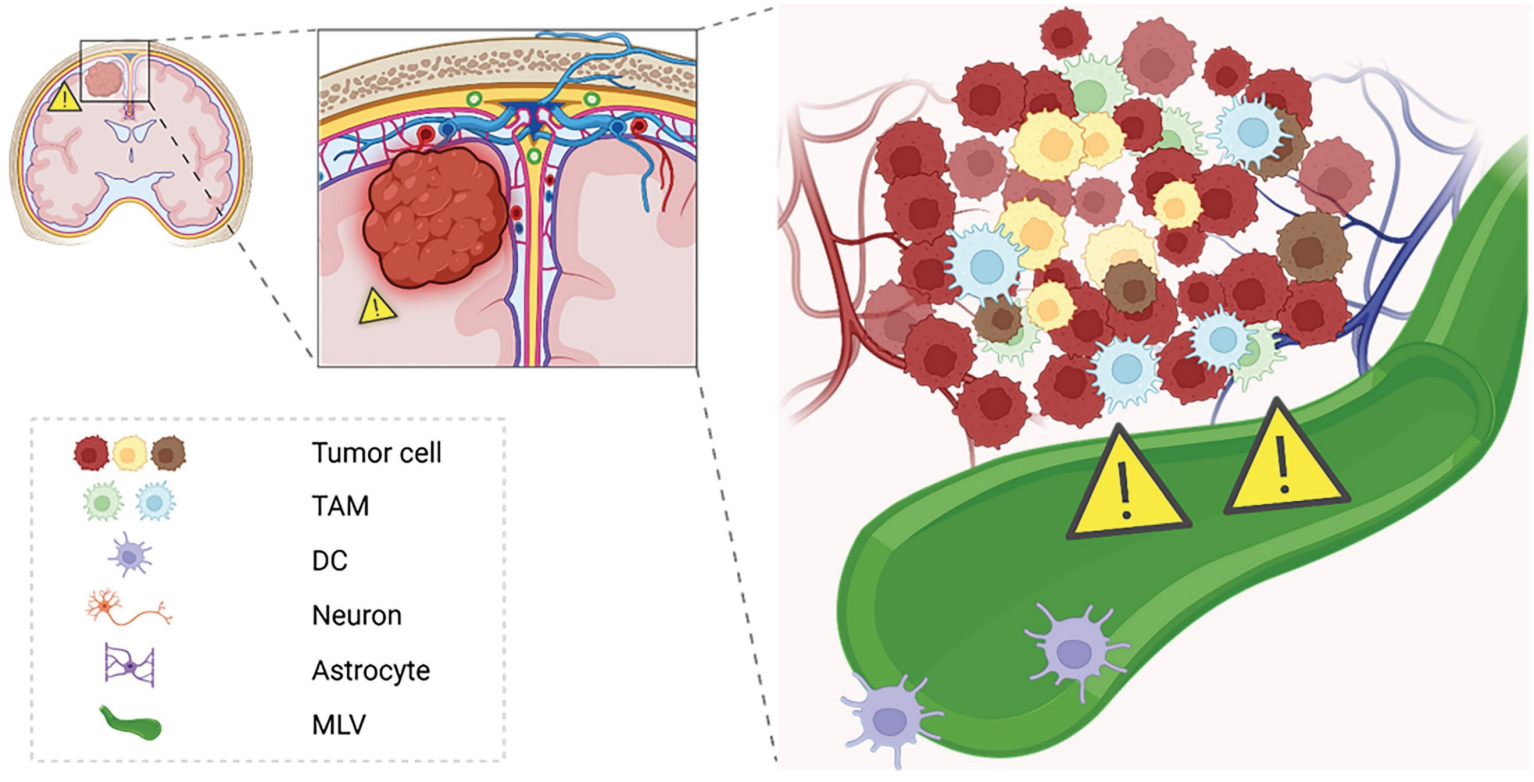
** Immunological potential of meningeal lymphatics under GBM states.** This schematic illustrates the dual role of meningeal lymphatics in GBM pathology. In the tumor state, reduced CSF outflow and exacerbated cranial hypertension impair the immunological functions of meningeal lymphatics. These vessels, while serving as pathways for CSF drainage, also facilitate antigen presentation and immune activation by transporting tumor-derived antigens to deep cervical lymph nodes. However, the pathological state compromises their capacity for immune surveillance, potentially allowing immune evasion.

Overall, MLVs represent a double-edged sword in glioblastoma immuno-oncology (Figure 6). On one hand, they may support antigen clearance and immune surveillance, potentiating the effects of checkpoint blockade therapies. On the other, aberrant lymphangiogenic remodeling could theoretically aid tumor progression or immune evasion under certain conditions. A deeper mechanistic understanding of MLV-tumor interactions will be essential for designing strategies that enhance their anti-tumor roles while minimizing potential adverse effects, opening new avenues for immunotherapy optimization in GBM.

## 5. Therapeutic potential of targeting meningeal lymphatics in glioblastoma

### 5.1 Limitations in current GBM therapies

**Figure 7:**
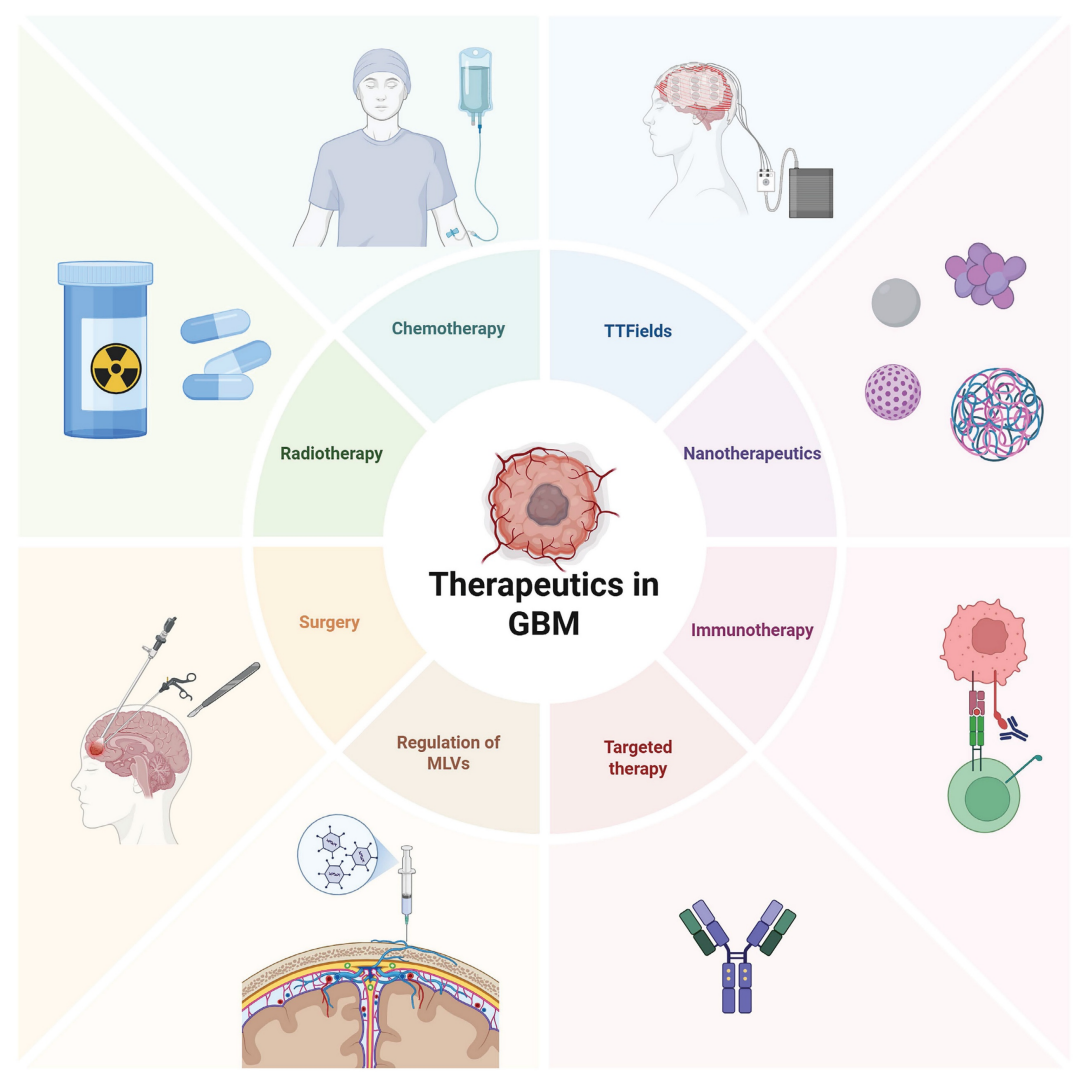
** Current treatment strategies in GBM.** Current glioma treatment presents a diversified and integrated model. Surgical operation remains the core approach, with intraoperative navigation and electrophysiological monitoring being utilized to expand the resection range while protecting functional areas. Postoperative standardized chemotherapy mainly uses temozolomide, combined with conformal intensity-modulated radiotherapy. However, issues such as drug resistance and long-term cognitive impairment remain prominent. Among the emerging new strategies in recent years, immunotherapy, such as immune checkpoint inhibitors and CAR-T therapy, is undergoing clinical trials, and targeted drugs for specific gene mutations (such as IDH1 inhibitors) have entered the application stage. Tumor treatment fields (TTFields) demonstrate unique advantages by interfering with cell division through low-frequency alternating electric fields. In the frontier field, nanoparticle drug delivery systems can cross the blood-brain barrier to deliver drugs specifically, while regulating the function of MLVs provides treatment from the aspects of metabolic clearance and immune microenvironment regulation.

GBM remains one of the most challenging cancers to treat due to several factors, including the BBB, the TME, and the highly invasive nature of the tumor. GBM experiences limited immune surveillance within the brain, partly due to molecular restrictions and the limited ability of immune cells to cross the BBB 124. Additionally, the tumor microenvironment is highly heterogeneous, with different niches fostering drug resistance and immune evasion. Within these niches, tumor cells undergo clonal selection, leading to mutations and the emergence of treatment-resistant subpopulations. These challenges limit the effectiveness of classic therapeutic approaches, including surgery, chemotherapy, and radiation therapy (Figure 7) 125-127.

Currently, surgical resection is the cornerstone of GBM management. It provides rapid decompression to alleviate intracranial hypertension and allows for histopathological and molecular characterization, including IDH mutation and MGMT promoter methylation, which guide postoperative precision therapy 128. However, due to the diffusely infiltrative growth of GBM, complete resection is rarely feasible. Residual tumor cells often remain within or beyond the resection margins, leading to frequent local recurrence 129. In cases where the tumor invades functional areas, such as the language or motor cortex, surgical intervention poses a significant risk of neurological deficits 130.

Given the infiltrative growth pattern of gliomas, postoperative adjuvant therapies, including radiotherapy and chemotherapy, are often necessary to delay recurrence 131. Radiotherapy precisely targets residual tumor areas with high-energy beams, effectively controlling postoperative lesions. Nevertheless, prolonged radiotherapy may induce radiation necrosis or cognitive dysfunction in brain tissue, necessitating strict dose control 132. In chemotherapy, the Stupp protocol combining temozolomide with radiotherapy has become the standard treatment for high-grade gliomas, with the advantage of penetrating the blood-brain barrier 133. However, side effects such as bone marrow suppression and the development of drug resistance limit long-term benefits for some patients 134.

In recent years, targeted therapies and immunotherapies have expanded the therapeutic landscape. For example, the anti-angiogenic agent bevacizumab can reduce tumor-related brain edema but offers only transient benefits and is costly, with responses largely limited to VEGF-high subtypes 135, 136. Immunotherapy approaches, including immune checkpoint inhibitors and CAR-T cell therapy, have demonstrated potential in activating anti-tumor immune responses. However, challenges such as the blood-brain barrier and the complexity of the immune microenvironment have impeded consistent therapeutic outcomes, and ongoing clinical trials continue to explore optimized protocols 137. Additionally, tumor electric field therapy, which inhibits tumor cell division through non-invasive physical intervention, significantly extends survival when combined with the Stupp protocol. Yet, the requirement for long-term device use imposes significant compliance demands on patients 138, 139. Despite the emergence of multimodal treatment strategies, the immune evasion mechanisms of GBM remain unresolved, underscoring the urgent need for innovative therapeutic approaches.

### 5.2 Therapeutic potential of MLVs in glioblastoma

MLVs have emerged as promising therapeutic targets in GBM due to their dual functionality in CSF drainage and immune modulation. As upstream components of the dCLNs, MLVs play an essential role in linking the intracranial and peripheral immune systems. Multiple studies have demonstrated that dCLNs act as immunological gateways for brain-derived antigens. Liposomes or tracers injected into dCLNs can reach the meninges, brain parenchyma, and even tumor sites. For instance, photodynamic liposomes delivered to the dCLN have been shown to shrink glioma lesions in rats under near-infrared laser irradiation 140. This anatomical link also enables immune cross-communication: antigens introduced into the brain parenchyma or subarachnoid space can stimulate specific antibody production in the dCLNs 141, 142.

MLVs, as the upstream conduits of these lymph nodes, offer unique opportunities for targeted drug delivery and immune activation. In a study of photodynamic efficacy of glioblastoma in rats 143, researchers found that the sensitivity of 6- and 24-month-old rats to the therapeutic effects of GBM was correlated with the age, which in turn was correlated with the function of the MLVs. The aging brain is characterized by a decline in the function of the MLVs, resulting in a decrease in the drainage of CSF, which leads to a decrease in the efficacy of photo-stimulation of the MLVs to inhibit GBM 91, 144. This emphasizes the importance of maintaining MLV integrity to optimize treatment outcomes, especially in elderly patients.

**Figure 8:**
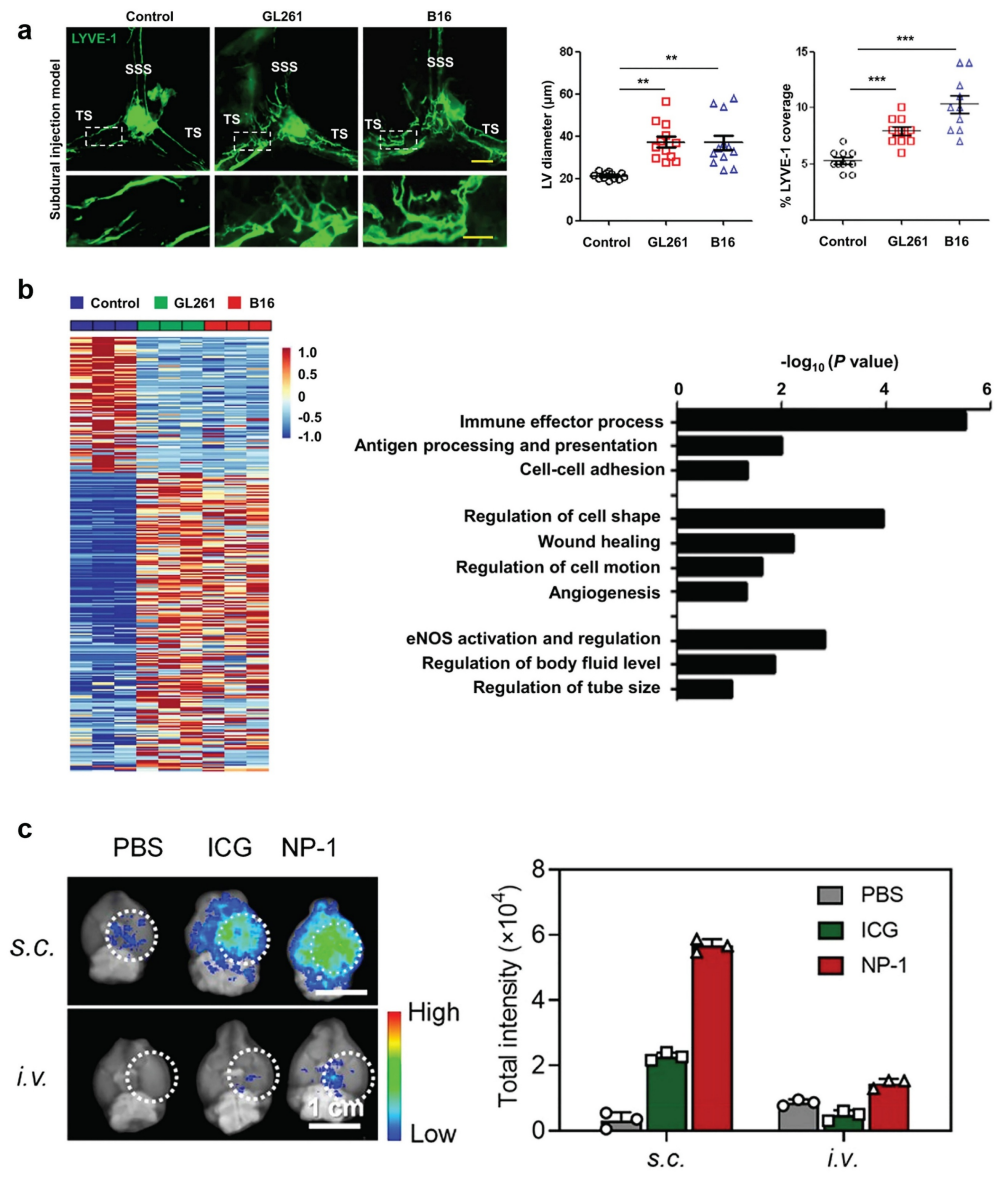
** Current exploration of MLVs in the treatment of GBM. (A)** Left: representative meningeal LYVE-1 staining 1 week after subdural injection of GL261 or B16 cells into WT mice. Right: quantification of the diameter (n = 12) and percentage area (n = 10) of LYVE-1^+^ MLVs around the TS. Scale bars, 500 µm in wide-fields; 100 µm in insets. **(B)** Left: heat map of differentially expressed genes (Up, 219; Down, 100; power > 0.4). Right: gene sets involved in lymphatic remodeling, fluid drainage, as well as inflammatory and immunological responses as shown by the representative upregulated pathways in GL261 tumor-associated and B16 tumor-associated MLECs compared to control MLECs. Reproduced with permission from Ref. 103. Available under a CC-BY 4.0 license. Copyright 2020, Center for Excellence in Molecular Cell Science, CAS. **(C)** Distribution of bare ICG and NP-1 in the brain of glioblastoma-bearing mice 24 h post-s.c. or i.v. injection. Dotted white circles outline tumor sites. Reproduced with permission from Ref. 150. Copyright 2020, American Chemical Society.

Under pathological conditions, MLVs retain functional drainage capabilities and undergo tumor-induced lymphangiogenesis, proved by fluorescence images involving in remodeling (Figure 8A) 104. By transporting CSF, immune cells, and tumor antigens to dCLNs, MLVs not only alleviate cranial hypertension but also facilitate antigen presentation, activating T cells and enhancing anti-tumor immune responses (Figure 8B). These processes collectively contribute to slowing disease progression and improving therapeutic outcomes 104, 145.

One promising avenue for targeting MLVs involves the use of VEGF-C to enhance lymphangiogenesis and immune modulation 116. Prophylactic VEGF-C used in GBM models have demonstrated significant benefits, including enhanced lymphatic function, improved CD8^+^ T cell activation, and synergistic effects with immune checkpoint inhibitors such as anti-PD-1 and anti-CTLA-4 therapies 144, 145. For instance, exogenous VEGF-C promotes MLV function and proliferation, enhancing CCL21/CCR7 signaling and boosting the efficacy of checkpoint inhibition therapies. Conversely, chemoablation of dorsal MLVs, which reduces DC drainage to dCLNs, significantly diminishes the anti-tumor effects of these therapies 104, highlighting the essential role of MLVs in immune regulation. Additionally, VEGF-C has been shown to sensitize GBM to radiotherapy by enhancing meningeal lymphatic proliferation through the VEGF-C-CCL21 pathway 17. These findings suggest a synergistic potential between MLVs-targeted and conventional treatments.

In GBM, Limited T cell infiltration and the restrictive nature of the BBB remain major barriers to GBM immunotherapy 98. Current research primarily focuses on overcoming the BBB to deliver therapeutic agents directly to GBM sites 146-149. Beyond immune regulation, MLVs provide an alternative route for transporting immunogens and therapeutic agents into the CNS. For example, Zhao *et al.* 150 demonstrated that subcutaneous injection of indocyanine green (ICG)-loaded PLGA nanoparticles near cervical lymph nodes resulted in a 44-fold increase in brain accumulation compared to intravenous delivery (Figure 8C). Besides, preclinical studies have shown that drugs injected into cervical lymph nodes can successfully reach the brain via lymphatic pathways, bypassing the BBB 88, 105, 151. The therapeutic potential of MLV-based drug delivery is further supported by clinical trials. Clinical studies further support this strategy. A phase III trial evaluating the dendritic cell vaccine DCVax-L showed improved patient outcomes when antigen presentation was enhanced through lymphatic delivery systems 78, 105. These vaccines have shown promise in activating systemic immune responses and strengthened hope in improving patient survival. Therefore, by combining lymphatic delivery systems with immune-modulatory therapies or other conventional therapies, future approaches may achieve synergistic effects, overcoming some of the challenges posed by GBM's highly immunosuppressive TME.

### 5.3 Challenges for MLV-targeted therapies

Although targeting MLVs holds therapeutic promise in GBM, several anatomical, physiological, and oncological challenges must be addressed for clinical translation. Compared to peripheral lymphatic systems, MLVs in the CNS have smaller luminal diameter and less tissue coverage compared to peripheral lymphatics, limiting their capacity for fluid drainage and immune activation 16. Aging further complicates MLV function: basal MLVs, structurally optimized for CSF clearance, undergo progressive degradation and edema with age, leading to impaired CSF outflow and exacerbated intracranial hypertension, which is especially relevant in elderly GBM patients 15, 16, 144, 152, 153. In contrast, dorsal MLVs exhibit greater susceptibility to tumor-induced remodeling. In glioma models, disruption of dorsal MLVs reduces lymphatic remodeling, suggesting that distinct MLV subtypes play context-specific roles in tumor progression 104, 154. These findings highlight the anatomical and functional heterogeneity of MLVs, and suggest that future therapies must be tailored to the anatomical context and age-related status of each patient.

The plasticity of MLVs offers both opportunities and challenges for therapeutic intervention. For example, VEGF-C-induced lymphangiogenesis can modestly increase MLV diameter and improve drainage capacity, which may enhance immune cell trafficking and antigen presentation 116. However, this same pathway may be co-opted by tumor cells for dissemination. Glioma cells may interact with MLVs via integrin-mediated adhesion or degrade surrounding extracellular matrix through MMPs, enabling migration along lymphatic channels 119, 122, 123. Although extracranial metastases are rare in GBM, the theoretical risk of intracranial lymphatic dissemination via remodeled MLVs remains a concern, particularly as pro-lymphangiogenic therapies are integrated into immunotherapy regimens. Thus, a careful balance must be struck between augmenting immune surveillance and avoiding unintended pro-tumor consequences.

Overall, the dualistic nature of MLVs, functioning as both immune facilitators and potential metastatic conduits, necessitates nuanced therapeutic strategies. A deeper mechanistic understanding of MLV remodeling, aging-related changes, and their interaction with the TME is essential. Strategies that selectively enhance the anti-tumor functions of MLVs while minimizing their pro-tumor effects could pave the way for novel therapeutic approaches. By addressing these multifaceted challenges, the potential of MLVs to reshape GBM treatment paradigms may be fully realized.

## 6. Conclusions

MLVs represent a promising frontier in GBM research, offering novel insights into CSF drainage, immune surveillance, and TME remodeling. Their dual role as both immune regulators and pathways for CNS drainage positions MLVs as potential therapeutic targets for GBM. Recent technological progress has significantly advanced our ability to visualize MLV structure and function. Techniques such as immunofluorescence, magnetic resonance imaging, electron microscopy, and photoacoustic imaging have all contributed unique insights. For example, high-resolution immunofluorescence can delineate fine vessel structures ex vivo, while contrast-enhanced MRI modalities such as 3D T2-FLAIR and dynamic contrast-enhanced (DCE) sequences allow *in vivo* monitoring of lymphatic drainage dynamics and clinical translation potential 104, 155. However, most techniques remain limited by either resolution, invasiveness, or inability to provide dynamic, three-dimensional mapping of MLVs in small animal models 97, 156. Notably, dual-contrast functional photoacoustic microscopy (DCF-PAM) has demonstrated the ability to capture dynamic three-dimensional MLV trajectories *in vivo*, distinguishing their spatial relationship with cerebral vessels using indocyanine green (ICG)-labeled tracers 101. These imaging advances are essential for evaluating MLV-targeted therapies and understanding their mechanistic impact in real time.

Despite these advances, significant challenges remain. The structural heterogeneity of MLVs, along with their tumor-induced remodeling, underscores the need for a deeper understanding of their interactions with immunosuppressive TME. GBM leverages multiple immune escape mechanisms, limiting the ability of immune cells to infiltrate the tumor and weakening systemic immune responses. Addressing these challenges requires an intricate balance between enhancing MLV-mediated immune activation and mitigating their potential to facilitate tumor cell dissemination. Innovations in targeting the molecular pathways governing MLV remodeling, as well as refining their immune functions, will be essential to fully exploit their therapeutic potential.

The future of MLV-targeted therapies lies in their integration with existing and emerging treatment modalities. Combining MLV-based approaches with immune checkpoint inhibitors, radiotherapy, and advanced drug delivery systems offers a unique opportunity to overcome the limitations of the BBB and reinvigorate anti-tumor immunity. Additionally, the development of novel imaging technologies could enable real-time monitoring of MLV dynamics, allowing researchers and clinicians to fine-tune interventions and better understand treatment responses. Furthermore, insights gained from GBM research could have implications beyond oncology, providing a framework for exploring the role of MLVs in other CNS disorders.

In conclusion, MLV-targeted therapies represent a transformative opportunity for treating GBM and other challenging CNS diseases. By addressing current barriers and leveraging their unique properties, MLVs could redefine our approach to CNS disorders, bridging the gap between foundational research and clinical application. With continued interdisciplinary efforts, the therapeutic potential of MLVs can be harnessed to offer new hope for patients facing this devastating disease.

## Abbreviations

GBM: Glioblastoma
hGBM: human GBM
mGBM: mouse GBM
CNS: central nervous system
BBB: blood-brain barrier
MLV: meningeal lymphatic vessel
CSF: cerebrospinal fluid
VEGF-C: vascular endothelial growth factor-C
GSC: glioma stem cell
NSTC: non-stem tumor cell
TME: tumor microenvironment
TAM: tumor-associated macrophage
Mo-TAM: monocyte-derived TAM
BMDM: bone marrow-derived macrophage
TGF-β1: transforming growth factor-β1
MHC: major histocompatibility complex
IL: interleukin
IDH: isocitrate dehydrogenase
ADM: adrenomedullin
UMAP: uniform manifold approximation and projection
scRNA-seq: single-cell RNA
NK: natural killer
DC: dendritic cell
VRS: virchow-robin space
PVS: paravascular space
ISF: interstitial fluid
AQP4: aquaporin-4
MRI: magnetic resonance imaging
SMC: smooth muscle cell
SSS: superior sagittal sinus
TS: transverse sinus
dCLN: deep cervical lymph node
sCLN: superficial cervical lymphatic node
ECM: extracellular matrix
MMP: matrix metalloproteinase
TMZ: temozolomide
PD-1: programmed cell death protein-1
CTLA-4: cytotoxic T-lymphocyte antigen 4
CCL21: chemokine ligand 21
CCR7: chemokine receptor 7
ICG: indocyanine green
PLGA: poly(lactic-co-glycolic acid)
HDC: histidine decarboxylase
HNMT: histamine N-methyltransferase
CV: cerebral vessels
DCF-PAM: dual-contrast functional photoacoustic microscopy
DCE-MRI: dynamic contrast-enhanced MRI
SUR: signal unit ratio
